# Berberine attenuates TNBS-induced colitis in mice by improving the intestinal microbiota

**DOI:** 10.3389/fmicb.2024.1463005

**Published:** 2024-08-29

**Authors:** Chao Li, Xinxin Yin, Changpeng Xie, Jin Zeng, Chuan Song, Guibin Yang, Jinglei Zhang, Siai Chen, Panjian Wei, Ziyu Wang, Meng Gu, Wei Li, Juan An, Yuanming Pan

**Affiliations:** ^1^Department of Gastroenterology, Beijing Anzhen Hospital, Capital Medical University, Beijing, China; ^2^Department of Basic Medical Sciences, Qinghai University Medical College, Xining, China; ^3^Department of Gastroenterology, Aerospace Center Hospital, Peking University Aerospace School of Clinical Medicine, Beijing, China; ^4^Cancer Research Center, Beijing Chest Hospital, Capital Medical University/Beijing Tuberculosis and Thoracic Tumor Research Institute, Beijing, China

**Keywords:** berberine, intestinal microecology, colitis, probiotics, *Akkermansia muciniphila*

## Abstract

**Objective:**

To investigate the effects of berberine (BBR) as a treatment on intestinal microecological alterations and enteritis in mice produced by TNBS.

**Methods:**

There were seven mice per group: seven in the healthy group (Ctrl), seven in the TNBS-induced enteritis group (TNBS), and seven in the berberine treatment group (BBR). The mice were weighed, slaughtered after 7 days, and subjected to high-throughput intestinal microecological analysis by Illumina, as well as haematological detection and imaging evaluation of colon pathology.

**Results:**

The alterations in colon length, immune cell subpopulations, inflammatory factors, and intestinal microecology of mice induced by BBR were refined using a battery of experiments and observations. According to intestinal microecological studies, BBR can increase the number of bacteria, including *Lactobacillus*, *Verrucomicrobia*, *Bacteroides*, and *Akkermansia muciniphila*.

**Conclusion:**

BBR has a therapeutic effect on TNBS-induced colitis in mice, which is associated with modifications in immune cell subpopulations and intestinal microecology. It also offers a viable approach as a prospective probiotic (like *Akkermansia muciniphila*) to IBD therapy in clinical settings.

## 1 Introduction

The naturally occurring isoquinoline alkaloid berberine (BBR) is present in a variety of plants belonging to the genus *Berberis*. Although this substance has a long history in conventional medicine, recent studies have shown that it possesses a powerful pharmacological toolkit with the ability to exert antimicrobial, anti-inflammatory, anticancer, hypoglycemic, and lipid-lowering effects (Yan et al., 2015; Wang et al., 2020; Gong et al., 2021; Han et al., 2021). BBR provides a safety profile characterised by comparatively few side effects, despite its strong effects (Rauf et al., 2021). Its unique combination of safety and efficacy makes it a valuable addition to the pharmaceutical arsenal, especially in light of the growing emphasis on the advantages of natural substances.

With a growing reputation in recent years, BBR’s medicinal scope encompasses the core of intestinal disease and metabolic control. Its use has been documented in the treatment of metabolic diseases (Feng et al., 2019), a group of illnesses defined by changes in the body’s energy balance (Zhang et al., 2014). Similarly, its influence on illnesses of the intestines has brought it to the forefront of gastrointestinal study, including anything from basic absorption problems to intricate immunological reactions.

Berberine’s ability to treat inflammatory bowel diseases (IBD), such as Crohn’s disease (CD) and ulcerative colitis (UC), is a key component of its therapeutic promise (Cao et al., 2023). The gastrointestinal tract’s recurring, chronic inflammation serves as the foundation for many illnesses. Growing research indicates that dysbiosis, or an imbalance in the gut microbiota, and abnormalities in the host’s immune system play critical roles in the development and course of IBD, even if the exact causes and mechanisms of the disease are still unknown (Zhang and Li, 2014). Thus, an imbalance in the immune cells’ and gut microbiome’s interaction may have an impact on intestinal mucosal homeostasis. A balance in ILCs levels is, therefore, important for intestinal homeostasis, with abnormal levels potentially contributing to colitis in patients with IBD. Moreover, the complex balance between ILCs and Th17 cells, regulated by AHR and the symbiotic flora, is also essential for intestinal homeostasis (Luo et al., 2022). Berberine is a candidate that holds great potential for intervention because of its ability to reduce inflammation and balance the gut microbial ecosystem (Zhu et al., 2022).

Furthermore, IBD is a complex condition that is linked to a number of variables, including stress, mood, mental illness, and environmental and genetic factors in addition to microbiome abnormalities (Fairbrass et al., 2022). Intestinal microbiota composition is impacted by physical stress. Through the HPA axis, stress can raise circulating CRF and glucocorticoid levels. It can even permanently alter CRF, which can alter the function of the intestinal mucosal barrier and the makeup of microbes, resulting in gastrointestinal disorders (Zhang H. et al., 2023). IBD is linked to multiple signalling pathways, including the PTEN-PI3K-Akt-mTOR signalling pathway. Patients with colitis had significantly higher levels of PTEN and significantly lower levels of miR-21 in their intestinal mucosa. According to Wang Z. et al. (2022) transfection of miR-21-5p mimics can drastically down-regulate PTEN expression in PBMCs and up-regulate the PI3K-Akt-mTOR signalling pathway and downstream pathways. Involved in numerous pathophysiological processes, including angiogenesis, vascular permeability, and inflammatory responses, the VEGF and EGFR signalling pathways are linked to inflammation. Pro-inflammatory cytokines like IL-6 and IL-8 are released when EGFR is activated, assuming that VEGF overexpression increases the generation of IL-4 and IL-13 in mice (Peng et al., 2024).

The effectiveness of BBR in addressing the overt symptoms and the subtle histological harms of IBD has been supported by empirical research using animal models and clinical trials (Dong et al., 2022). It may have a multimodal therapeutic effect by suppressing pro-inflammatory signalling pathways (Wei et al., 2023), improving intestinal barrier integrity (Gong et al., 2017), and changing the composition of the gut microbiome (Li et al., 2020). Beyond IBD, BBR has adaptability that broadens its application to metabolic illnesses, including hypertension (Cai et al., 2021), diabetes (Zhang et al., 2020), obesity (Shou and Shaw, 2023), and gastro-functional disruptions such constipation and diarrhoea (Yue et al., 2019). However, there is still much to learn about the precise mechanisms of action and therapeutic targets of BBR. In light of this, this paper acts as a synthesis and a beacon, illuminating the therapeutic implications of BBR in the treatment of IBD and maybe other conditions, as well as pointing the way for future clinical application and the search for innovative pharmaceuticals.

This study confirmed that BBR can improve TNBS induced colitis. BBR can alleviate the pathological changes and disorders of intestinal flora and immune cells caused by TNBS. BBR regulates specific gut microbiota, of which AKK microbiota may be a key component in alleviating TNBS induced colitis.

## 2 Materials and methods

### 2.1 Experimental animals and berberine

Mice: Male C57BL/6N mice, aged 6–8 weeks and weighing between 20 and 25 g, were kept in the Laboratory Animal Centre at Tsinghua University’s Specific Pathogen Free (SPF) facility [License number: SYSK (Beijing) 2019-0044]. Maintaining regular housing conditions and maintaining a stable temperature range of 20–25°C and humidity levels between 40–70% was done for the animals. Under the product number #2086-83-1, Shanghai Yuanye Bio-Technology Co., Ltd. provided berberine (BBR).

### 2.2 TNBS induction and BBR treatment

**Figure 1:**
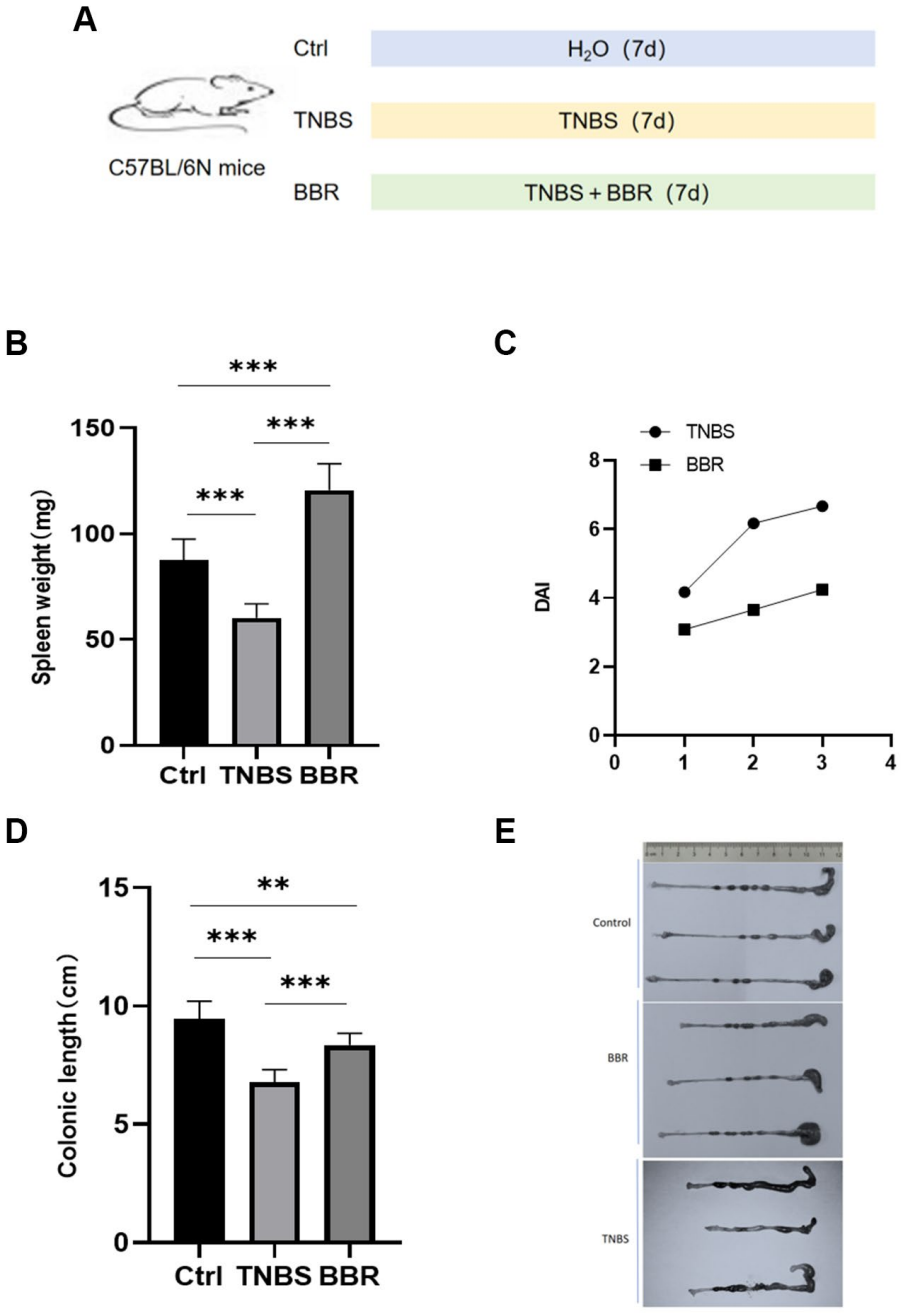
Changes of spleen weight, colon length and DAI value in mice. **(A)** Molding diagram. **(B)** Correlation test of spleen weight in each group. **(C)** Correlation test of DAI values between TNBS group and BBR group. **(D)** Correlation test of colon length in each group. **(E)** Comparison of colon length changes in each group (***: *p* ≤ 0.001, **: *p* ≤ 0.01, *: *p* ≤ 0.05, ns: *p* > 0.5).

The mice were split into three groups at random, consisting of seven animals each. In order to cause acute colitis, the second group was given 2,4,6-trinitrobenzene sulfonic acid (TNBS) treatment. To do this, 3.5 mg of TNBS was diluted in 100 μL of 30% ethanol, and a catheter measuring approximately 3 cm was introduced through the anus into the mice’s colon. BBR (100 mg/kg) (Li et al., 2016) was given intragastrically every day to the third group after being dissolved in sterile water (Figure 1A).

### 2.3 Model preparation and pathophysiological

Identification body weight, stool consistency, and faecal bleeding are recorded daily, and the colitis’s activity is indicated by the disease activity index (DAI) scoring system. The degree of weight loss, faecal consistency, and faecal bleeding are all included in DAI. The sum of the individual grades for the three aforementioned indications yields the final score. In terms of weight loss, 0 represents no change, 1 represents less than 5%, 2 represents 6–10%, 3 represents 11–20%, and 4 represents more than 20%. The following categories apply to stool: 0 = normal, 1 = soft and well-shaped, 2 = soft and particle-free, and 4 = diarrhoea. Blood: 0 indicates no blood, 1 indicates seepage of blood into the rectum, 2 indicates severe bleeding there, and 4 indicates blood visible on the fur (Chassaing et al., 2014). Following the completion of the model induction, the mice are rendered unconscious using 60 mg/kg of pentobarbital sodium and are then put to sleep by cervical dislocation. After the colon is gathered, its length is determined. Hematoxylin-eosin (HE) staining is performed on sectioned colon tissues that have been fixed in paraffin. Fresh colon tissues are fixed with electron microscope fixative in parallel, and preparation and photo analysis for scanning electron microscopy (Hitachi SU8020) and transmission electron microscopy (Japanese Electronics JEM1200EX) are carried out, respectively.

### 2.4 Intestinal microecology detection and bioinformatics

After **7** days, **10** faecal samples were chosen at random from the BBR, TNBS, and healthy (Ctrl) groups. 28.5 G of raw sequencing data were acquired during the sequencing process on the Illumina high-throughput sequencing platform. Every sample was added to the data analysis procedure for bioinformatics. Q.C., assembly, gene prediction, generation of non-redundant gene sets, annotation of gene functions, quantification of abundance at the gene, species, and functional levels, composition analysis, diversity analysis, multidimensional analysis, similarity analysis, difference analysis, functional analysis, and metabolite analysis were all included in the overall analysis.

### 2.5 Statistical analysis

Software called SPSS 28.0 was used to do the statistical analysis. Fisher’s exact test was used to analyse the count data, while the *t*-test was used to analyse the measurement data. Each group’s experimental data are shown as mean ± SD. One-way analysis of variance was used to compare the means between the groups. A substantial difference is considered statistically significant when *p* < 0.05.

## 3 Results

### 3.1 Changes in body weights and spleen weights

The mice had 3 days of continuous monitoring, and the weight changes were as follows: the mice in the healthy group gradually gained weight, the mice in the TNBS group gradually lost weight, and the mice in the BBR group lost weight gradually but less than in the TNBS group. The statistical significance of the difference between the two groups (*p* < 0.05) suggests that BBR has a positive impact on the weight loss of mice in the colitis model produced by TNBS. The spleen index, which is often correlated with the level of inflammation and anaemia, increased in mice given TNBS treatment as well (Figure 1B; Supplementary Table S1).

### 3.2 Disease activity index

The degree of weight loss, consistency of the stool, and level of faecal bleeding are used to calculate the disease activity index (DAI), which measures the activity of colitis in clinical settings. The BBR group’s disease activity index (DAI) is considerably lower than the TNBS-induced colitis group’s, according to the DAI, which was assessed in 4 mice (Figure 1C).

### 3.3 Changes in mouse colon length and pathological changes

**Figure 2:**
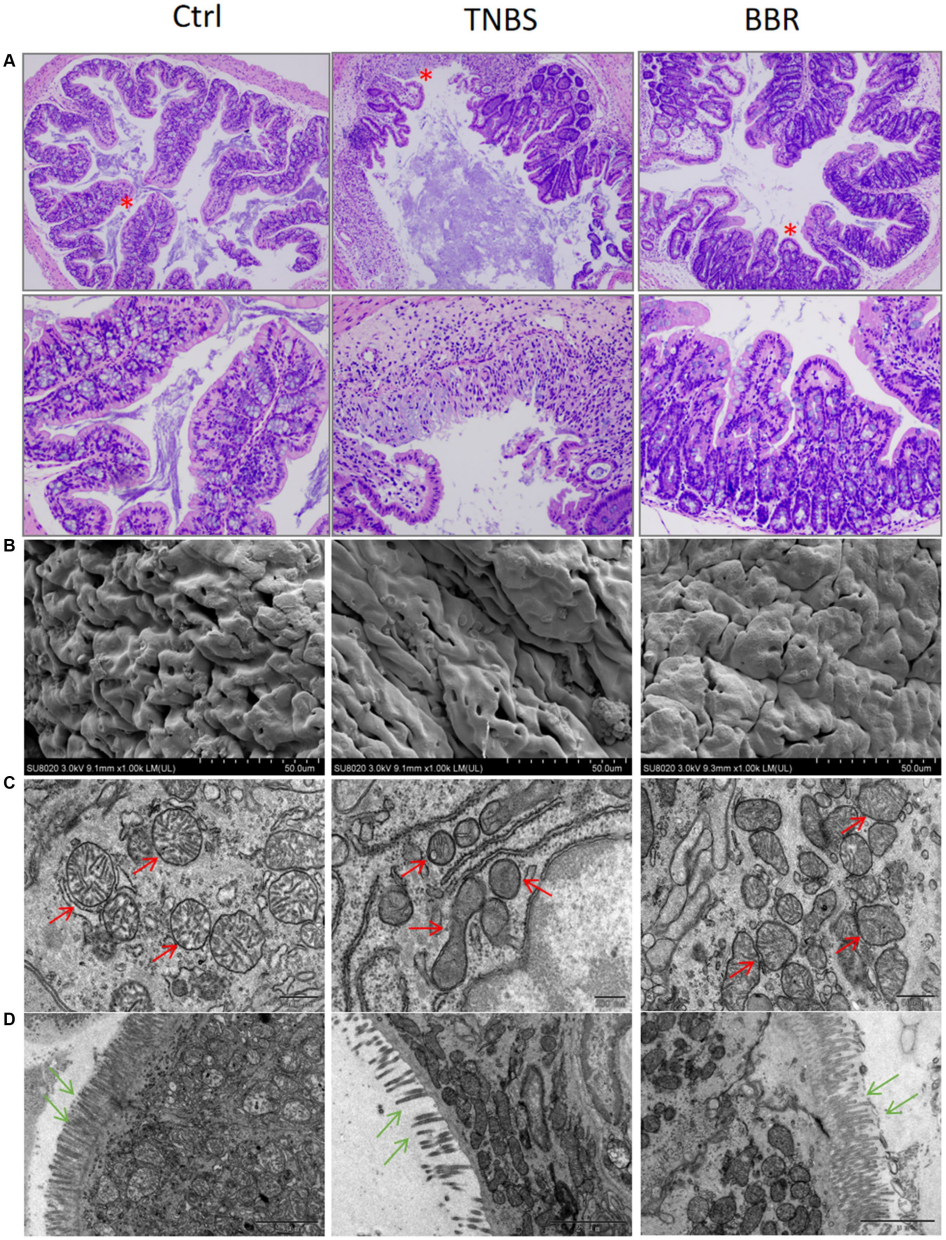
Protective effect of BBR on intestinal mucosal tissues in TNBS-induced colitis. **(A)** HE staining showing the effect of each group on the junction strands of intestinal mucosal tissues (up: 40×, down: 200×). **(B)** Scanning electron microscopy results of colon tissues of each group, BBR can reduce TNBS-induced intestinal mucosal oedema and erythrocyte exudation. **(C,D)** Transmission electron microscopy results of colon tissues of each group, BBR can ameliorate the mitochondria of the epithelial cells of the intestinal mucosa due to TNBS-induced crumpling and reduced numbers, and maintained the number and orderly arrangement of colonic microvillus structures.

Mouse colon length changes: BBR treatment group’s colon length was significantly longer than that of the TNBS group, and TNBS group’s colon length was significantly shorter than that of the normal group. This suggests that BBR partially mitigates the shortening of colons caused by TNBS (Figures 1D,E). Pathological alterations Using HE staining on the colon tissue of each group of mice, it was discovered that the intestinal glands were completely and neatly arranged, the mucosa showed no signs of inflammatory cell infiltration, and the muscle layer remained smooth and intact. The colon tissue structure of the control group and the normal group of mice was also found to be intact, without ulcers, and without damage. With a significant quantity of inflammatory cell infiltration and congestion, as well as cell shedding and necrosis in the upper layer of the mucosa, the colon tissue structure of the mice in the TNBS group has clearly been destroyed. The mice in the BBR group had mildly damaged colon tissue, clean villi, intact mucosa, no necrosis, and a minor degree of infiltration of inflammatory cells (Figure 2A).

### 3.4 Transmission and scanning electron microscopy of colon

Transmission electron microscopy (TEM, JEOL JEM1200EX) revealed that the crypt structure was damaged, the goblet cells were diminished, the epithelial cells’ mitochondria were condensed, and the epithelial cells in mouse colon tissue were disordered in the TNBS group. Scanning electron microscopy (SEM, Hitachi SU8020) revealed that the TNBS group had inflammatory cell infiltration, mucosal damage, etc., while the alterations in the BBR administration group were not evident (Figures 2B,C). Following BBR administration, it assisted in restoring the cellular mitochondria.

### 3.5 Hematological examination of mice

#### 3.5.1 Flow cytometry

**Figure 3:**
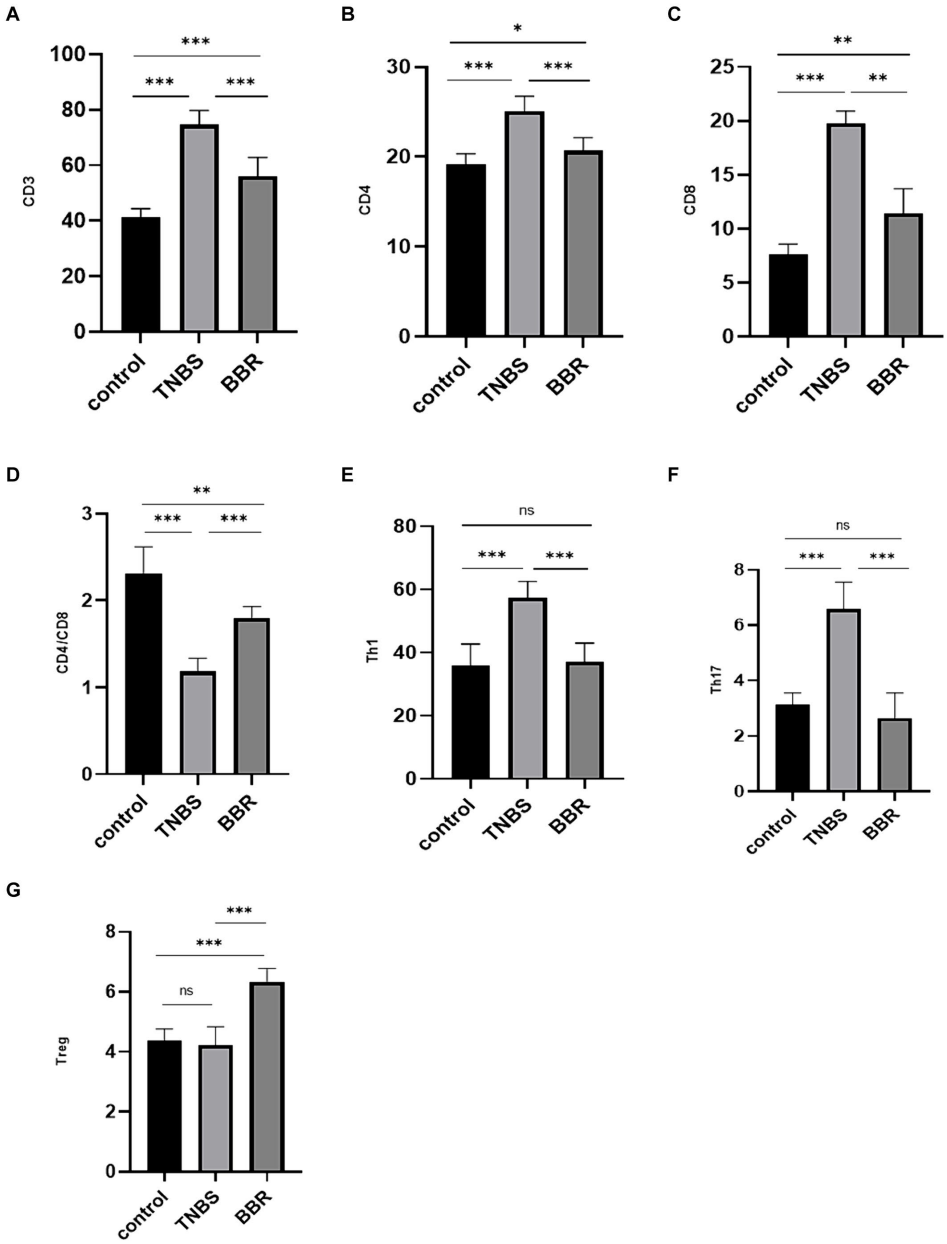
Correlation test of immune cell content in each group (***: *p* ≤ 0.001, **: *p* ≤ 0.01, *: *p* ≤ 0.05, ns: *p* > 0.5).

The contents of CD3 cells, CD4 cells, CD8 cells, Th1 cells, and Th17 cells are shown to significantly decrease following drug treatment, while the cell ratio of CD4/CD8 and Treg cells significantly increases following BBR treatment. Flow cytometry is used to identify the immunological subgroups at the serological level. This suggests that by controlling the intestinal immune response, BBR can lessen the symptoms of colitis (Figures 3A–G).

#### 3.5.2 Enzyme-linked immunosorbent assay

**Figure 4:**
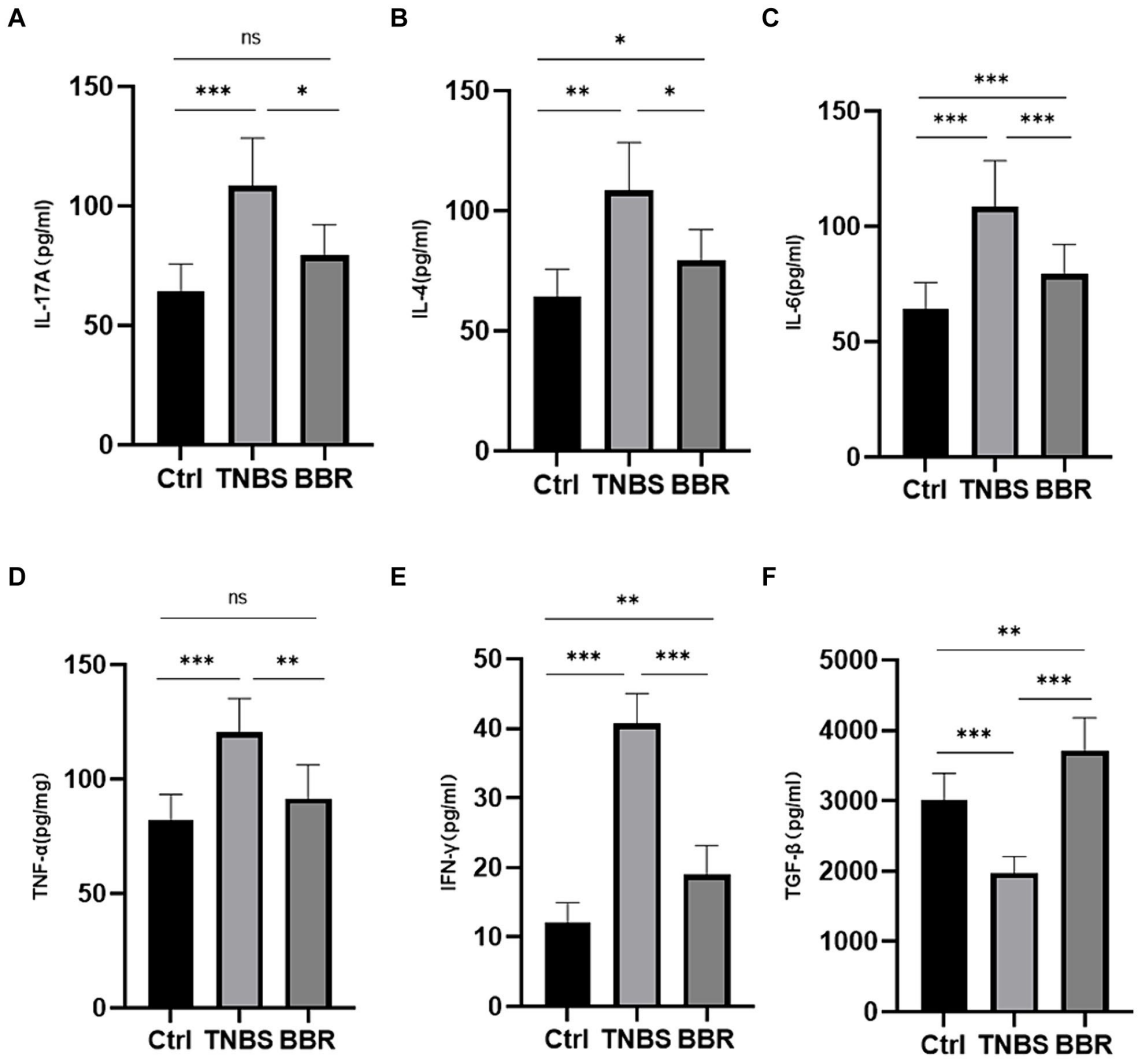
Correlation test of the expression levels of serum detection and inflammatory factors in each group (***: *p* ≤ 0.001, **: *p* ≤ 0.01, *: *p* ≤ 0.05, ns: *p* > 0.5).

Pro-inflammatory factor expression levels, including TNF-α, INF-γ, IL-4, IL-6, and IL-17A, were considerably greater in the TNBS group than in the normal group, suggesting successful modelling. In the BBR group, there was a decrease in the expression levels of TNF-α, INF-γ, IL-4, IL-6, and IL-17A. Furthermore, BBR has the ability to raise TGF-β levels, which exhibit anti-inflammatory properties (Figures 4A–F). These findings suggest that BBR can control the intestinal immune response by preventing the TNBS mice’s colon tissues from accumulating more cellular inflammatory factors.

### 3.6 Examining the gut microbiota in colitis-affected mice following BBR treatment

#### 3.6.1 Microbiota diversity and difference analysis

**Figure 5:**
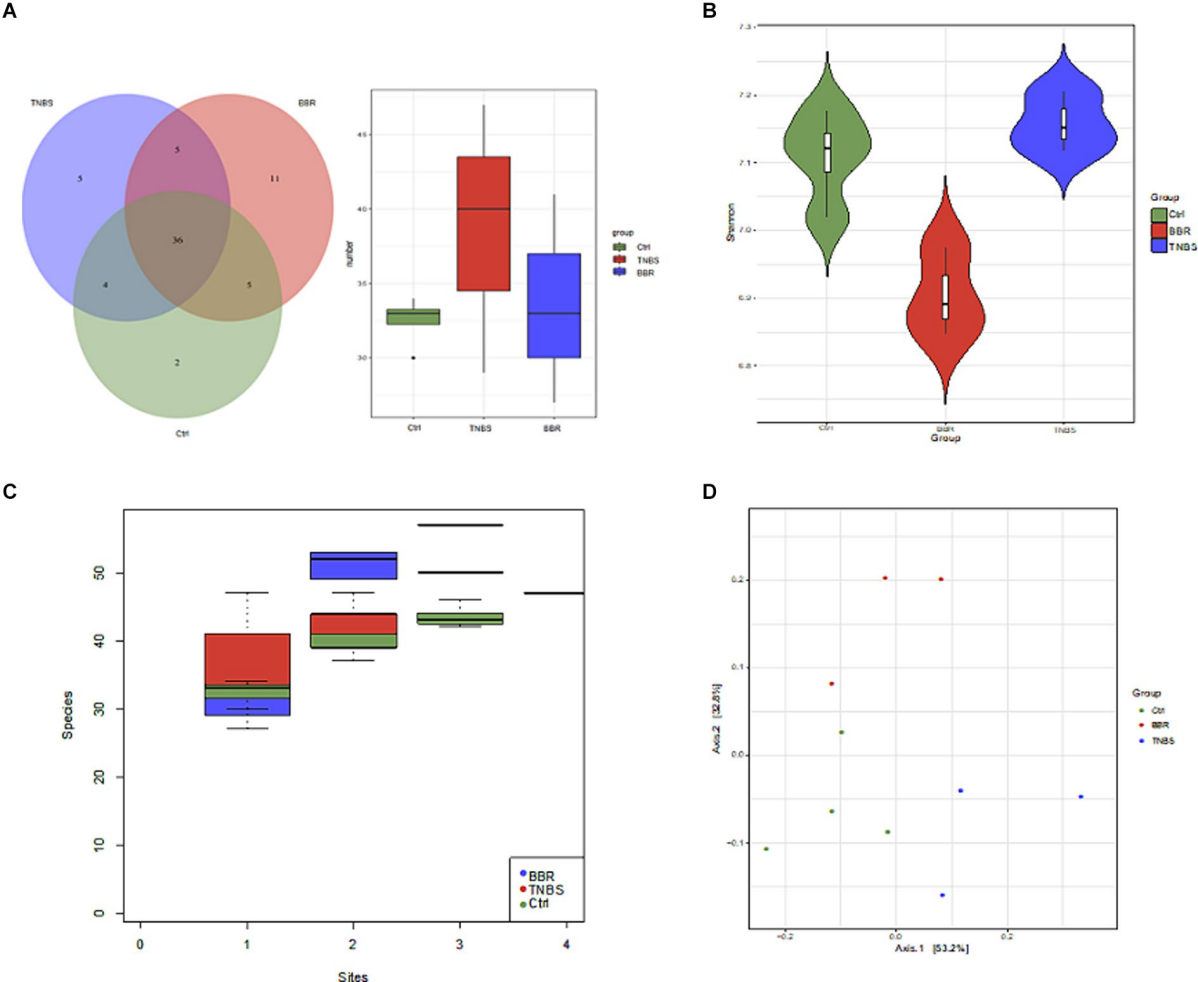
Diversity and difference analysis of microbiota between groups. **(A)** Venn diagram of genes between different groups. **(B)** Alpha diversity analysis. **(C)** Comparison of species accumulation curves between components. **(D)** PCoA analysis.

In between groups all original sequencing reads were processed with cutadapt software to remove the adapters and obtain clean reads, and all original metagenomic sequencing data were quality validated using MOCAT2 software (Supplementary Table S2). To exclude polluted host reads, the filtered reads were compared with the host genome using SOAPaligner, yielding clean, high-quality data. Next, in order to obtain scaftigs longer than 5,000 kb, gene assembly was carried out (Supplementary Table S3). Gene structure was predicted using MetaGeneMark; redundant genes were eliminated by clustering the predicted genes using CD-HIT, resulting in a non-redundant gene set of genes longer than 100 bp. Ten samples were used to create a non-redundant gene set, and several group genes were examined using Venn diagrams to highlight similarities and differences between the three (Figures 5A,B). After that, the intergroup species accumulation curve was compared to the differential microbiota, and it was shown that there was no statistically significant difference in microbiota diversity between the three (*p* ≤ 0.05) (Figure 5C). We utilised the Shannon index to represent the samples’ Alpha diversity in further diversity study. Following diversity analysis, the BBR group showed improvement whereas the TNBS group’s microbiota diversity and diversity were lower than those of the normal group (Figure 5C). Subsequent to the PCoA analysis (Figure 5D), a discernible distance was seen among the three groups, suggesting variations in the microbiota’s kinds and composition. These findings suggest that the intestinal microbiota’s structure changed following TNBS intervention, and that these alterations can be undone with BBR intervention.

#### 3.6.2 In colitis models, BBR enhanced the gut microbiota composition

**Figure 6:**
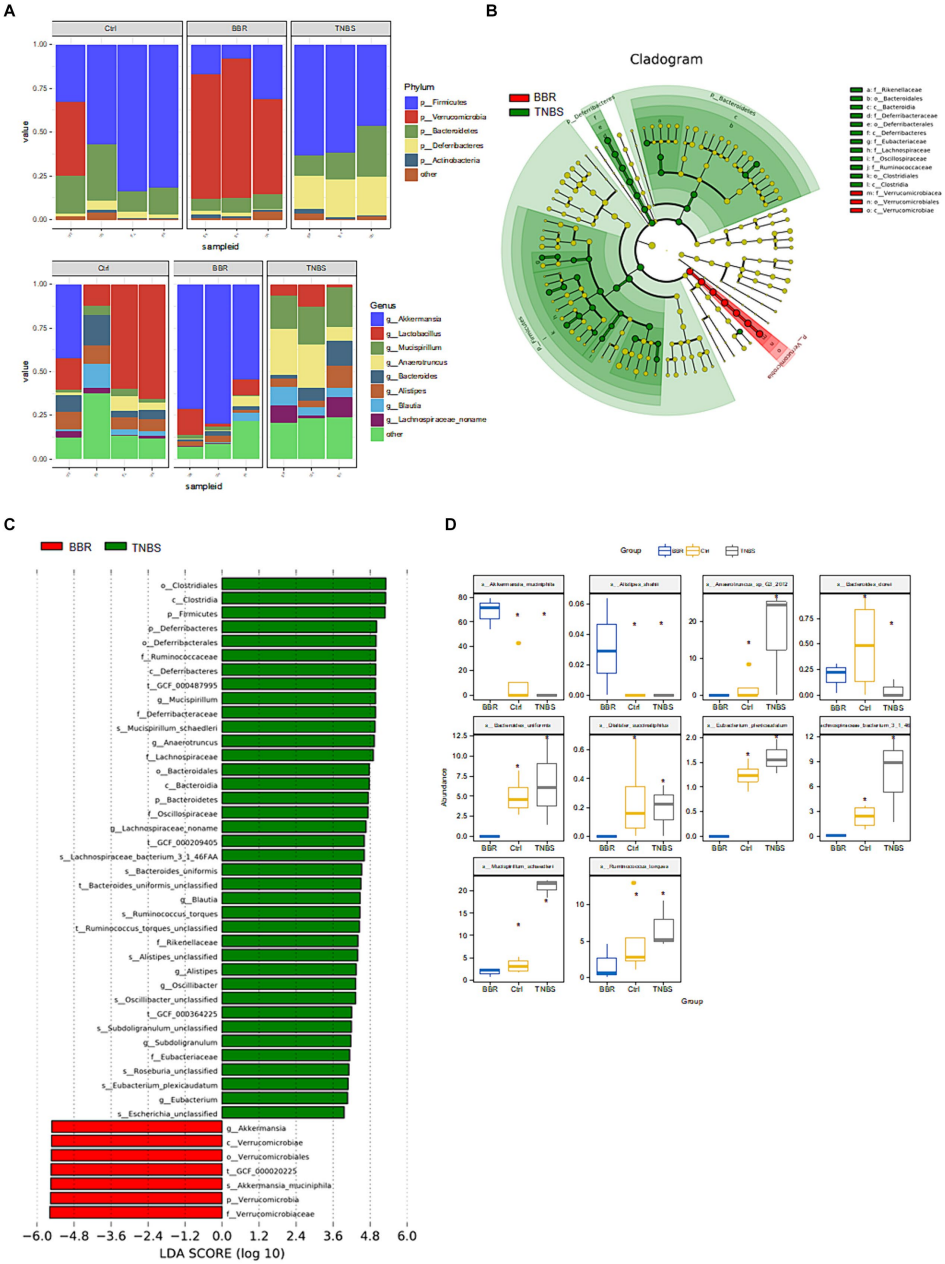
Composition and difference analysis of microbiota in each group. **(A)** Relative abundance composition at the phylum and genus levels in different groups. **(B,C)** Results of LEfSe analysis (LDA >4, *p* < 0.05). **(D)** Box comparison chart of significantly different bacteria (Top10).

Following phylum-level analysis of the gut microbiota, BBR was found to have significantly higher *Verrucomicrobia* abundance and significantly lower abundances of *Firmicutes*, *Bacteroidetes*, *Deferribacteres*, and *Actinobacteria* as compared to the TNBS group. Following BBR therapy, there was a considerable rise in the abundance of *Akkermansia* and a decrease in the abundance of *Lactobacillus*, *Mucispirillum*, *Anaerotruncus*, *Bacteroides*, etc. at the genus level (Figure 6A). LEfSe analysed the TNBS group and the BBR group; the results included an evolutionary branch map and an LDA value histogram (LDA >4, *p* < 0.05). There were 15 prokaryotic evolutionary branches on display. In the TNBS group, there was a larger abundance of *Bacteroidetes*, *Bacteroides*, *Deferribacteres*, *Lachnospiraceae*, and *Clostridiales*. In the BBR group, there was a considerable increase in the abundance of *Verrucomicrobia*, *Akkermansia*, and *Akkermansia muciniphila* (*Akk* bacteria) (Figures 6B,C). The two groups’ significantly different microorganisms were compared, in turn (Figure 6D).

**Figure 7:**
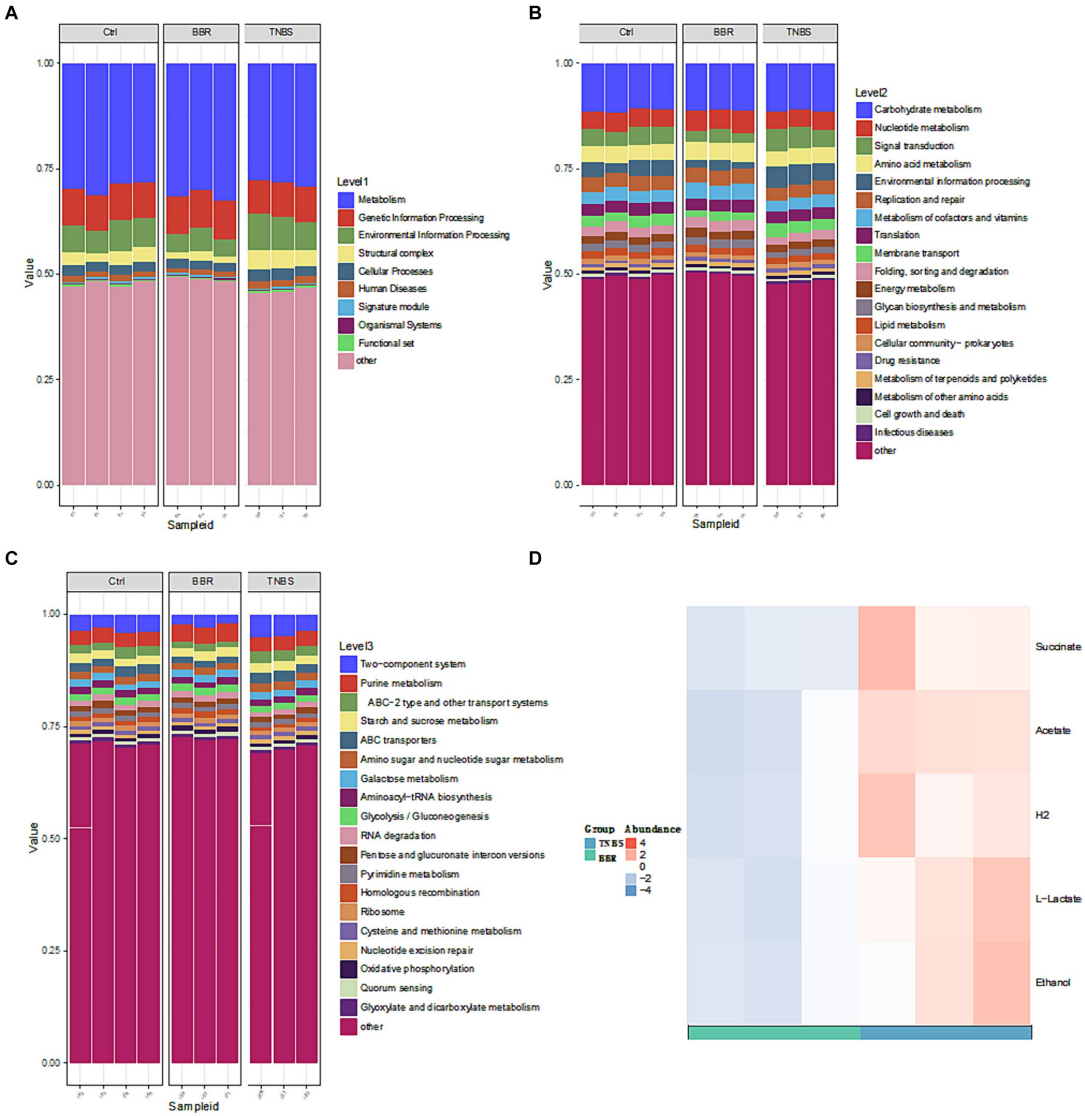
KO analysis of metabolite pathway enrichment and significant metabolite difference analysis between TNBS and BBR. **(A–C)** KO analysis of metabolite pathway enrichment at three levels. **(D)** Significant metabolite difference analysis between TNBS and BBR.

After that, a metabolic pathway enrichment map was produced using a three-level KEGG Orthology (KO) analysis (Figures 7A–C). To get differential metabolites, the three groups were compared individually based on the enhanced metabolic pathways. A total of 5 differential metabolites were found after a metabolite difference study between the TNBS group and the BBR group. The levels of succinic acid (succinate), acetate (acetate), H2, L-lactic acid (L-Lactate), and ethanol (ethanol) were higher in the TNBS group as compared to the BBR group (Figure 7D).

**Figure 8:**
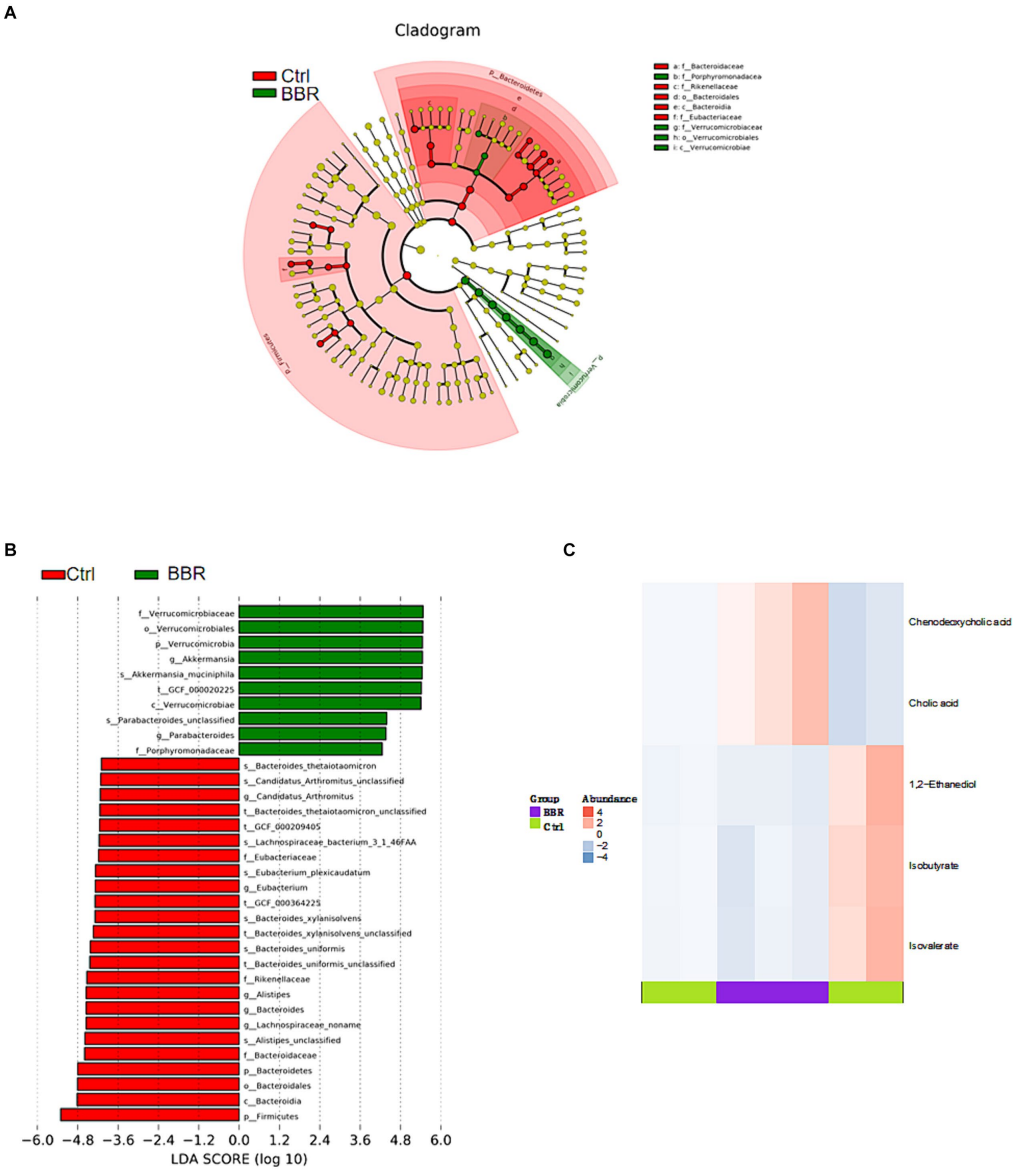
Comparison of microbiota composition and significant metabolic difference analysis between Ctrl group and BBR group. **(A,B)** Results of LEfSe analysis (LDA >4, *p* < 0.05). **(C)** Significant metabolic difference analysis between Ctrl group and BBR group.

The Ctrl group and the BBR group underwent LEfSe analysis (LDA >4, *p* < 0.05), which produced nine prokaryotic evolutionary branches. Among these, the BBR group had a larger abundance of *Verrucomicrobia* and *Akkermansia muciniphila* (*Akk* bacteria) compared to the Ctrl group’s higher abundance of *Bacteroidaceae*, *Bacteroides*, and *Eubacteriaceae* (Figures 8A,B). Five distinct differential metabolites were found after a metabolite difference study between the two groups was completed. While the BBR group had larger levels of chenodeoxycholic acid and catalic acid, the Ctrl group had higher levels of 1,2-ethanediol, isobutyrate, and isovalerate (Figure 8C).

**Figure 9:**
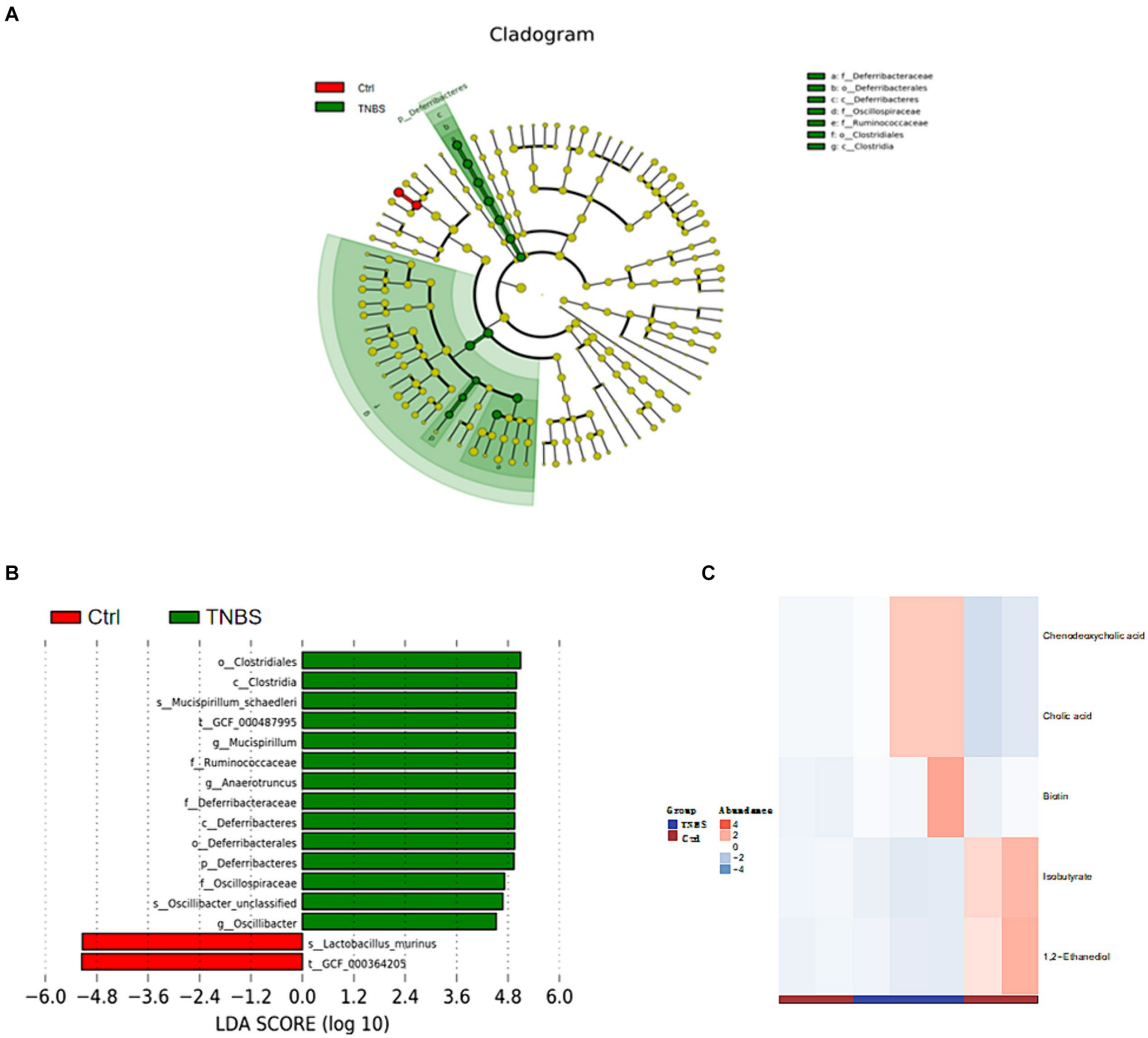
Comparison of microbiota composition and significant metabolic difference analysis between Ctrl group and BBR group. **(A,B)** Results of LEfSe analysis (LDA >4, *p* < 0.05). **(C)** Significant metabolic difference analysis between Ctrl group and BBR group.

The Ctrl group and TNBS group underwent LEfSe analysis (LDA threshold >4, *p* < 0.05), which produced six prokaryotic evolutionary branches. Of these, the Ctrl group had a larger abundance of *Lactobacillus murinus*, but the TNBS group had a higher abundance of *Deferribacteres* and *Clostridiales* (Figures 9A,B). Five distinct differential metabolites were found after a metabolite difference study between the two groups was completed. While the TNBS group had larger levels of chenodeoxycholic acid, catalic acid, and biotin, the Ctrl group had higher levels of 1,2-ethanediol and isobutyrate (Figure 9C).

## 4 Discussion

**Figure 10:**
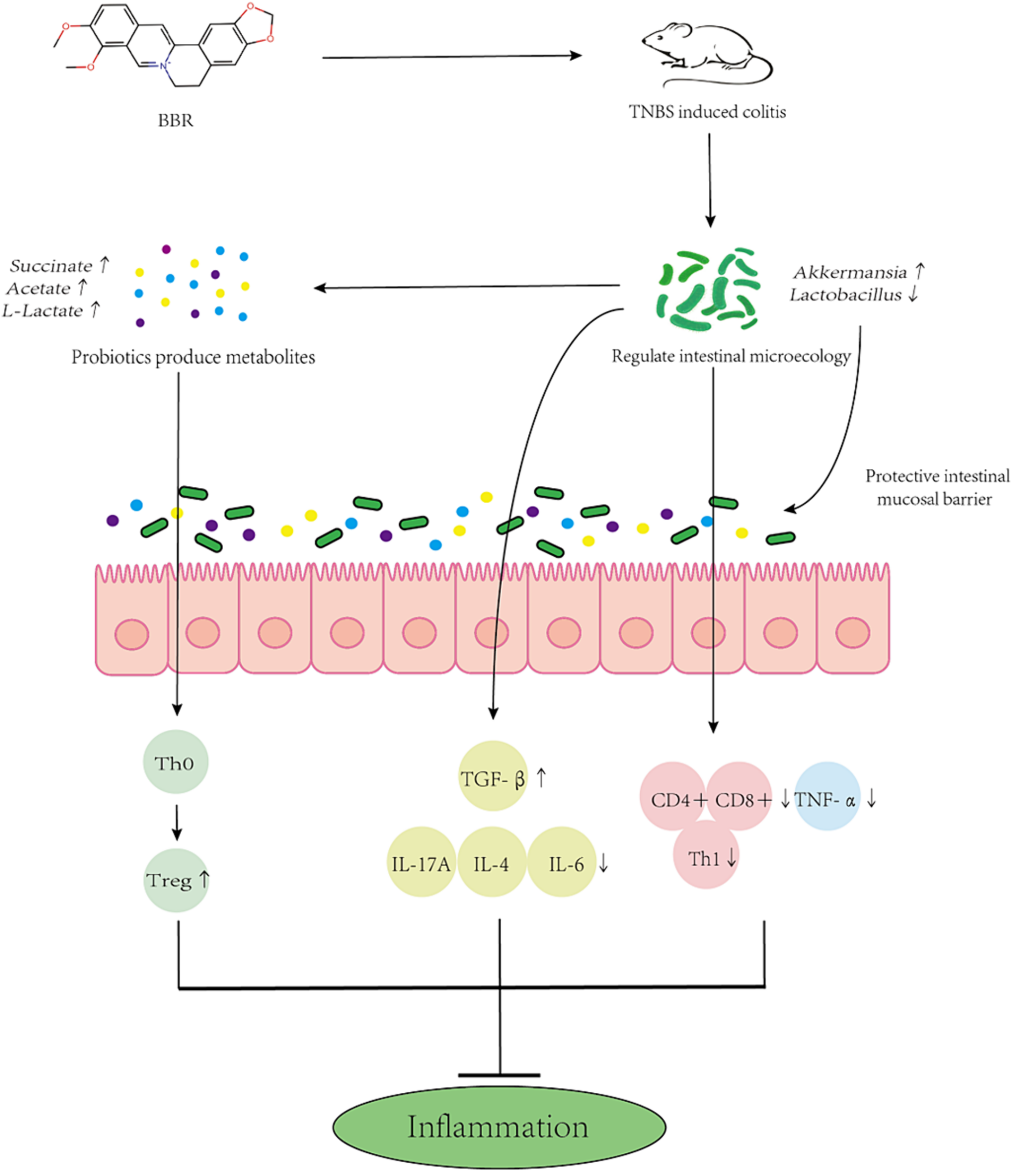
Mechanism simulation diagram. Schematic diagram of BBR maintaining the intestinal barrier and reducing inflammation by altering the composition of gut microbes and side populations of immune cells.

Research has indicated a correlation between the gut microbiota’s makeup and the severity of colitis. Dysbiosis of the intestinal microbiota, which results in an imbalance between beneficial and harmful bacteria and damages the intestinal microbial barrier, is a common feature of inflammatory bowel disease (IBD) (Ni et al., 2017). BBR possesses anti-inflammatory and antibacterial properties, which may make it useful in the treatment of intestinal disorders (Yang et al., 2024). Numerous chemicals, including polysaccharide components, control the makeup of gut bacteria. In contrast, 5-FU damages the gut immune system and alters microbial diversity, which is linked to the TLR4/MyD88/NF-kilb signalling pathway (Huang et al., 2023). Studies have shown that modification of rice resistant starch with heat-stable α-amylase and glucose amylase can increase the abundance of probiotics and reduce the number of harmful bacteria in the gut of T2DM mice. This study verified that BBR regulates the makeup of the gut microbiota to ameliorate colitis in mice. By suppressing the pathogenic microorganisms that cause enteritis, BBR can reduce the inflammatory response in the intestines. BBR, for instance, has the ability to inhibit and interfere with the synthesis and regulation of *Salmonella*-related proteins. BBR primarily has anti-inflammatory effects by preventing the production of inflammatory factors (like TNF-α, IL-8, etc.) and oxidative stress (Sujitha et al., 2018). As of right now, research on the mechanism of BBR in the treatment of colitis is still ongoing. According to earlier metabolomics research, BBR is associated with the citric acid cycle, amino acid metabolism, vitamin metabolism, and purine metabolism pathways. It can also reduce the inflammatory response in the intestines and preserve the integrity of the intestinal mucosal barrier (Wang H. et al., 2022). Intestinal microbiota metabolites (including tryptophan, BAs, and SCFAs) and improved intestinal barrier function may be the mechanisms by which BBR corrects the imbalance of intestinal microbial structure (Sun et al., 2023). We also investigated the relationship between BBR’s regulation of these metabolites and the alterations in gut flora. The findings of this study demonstrate that BBR can ameliorate colitis by reducing mouse weight, lengthening the colon, increasing spleen weight, reducing red blood cell exudation, improving the ridge structure in mitochondria, decreasing swelling, improving the orderliness and arrangement structure of microvilli in intestinal mucosal epithelial cells, enhancing the expression of pro-inflammatory factors to regulate the intestinal immune response, and regulating the composition of intestinal microbiota to alleviate colitis caused by TNBS. The intestinal microbiota’s quantity and composition differ significantly between colitis-affected and BBR-treated mice (Figure 10).

According to Al Ojaimi et al. (2020), TNBS can damage mitochondria and result in their collapse and deformation because the production of ROS in the jejunum activates UCP2, an antioxidant enzyme, and because mitochondrial uncoupling lowers the efficiency of energy production, which lowers the absorption of energy-dependent nutrients. Because BBR contains vitamin A, which increases mitochondrial transcription factors and NFRS—1 TFAM, efficiently protects colon mitochondria, prevents inflammation and colitis necrosis, and reverses the process of mitochondrial damage (Reifen et al., 2015). According to other research, demethyleneberberine reduces colitis in mice via controlling Th cell balance and blocking the NF-κB pathway (Chen et al., 2017). BBR reduced DNA fragmentation in hair cells and decreased the production of ROS while maintaining the potential of the mitochondrial membrane. Thus, by lessening the stress in mitochondria and promoting cell survival, BBR may function as an antioxidant against CP.

In order to treat colitis, BBR also primarily increases the abundance of *Akk*, *Firmicutes*, *Bacteroidetes*, *Actinomycetes*, and other bacteria. It also modifies the levels of acetic acid, succinic acid, H2, ethanol, and other metabolites. In conclusion, our findings demonstrate that BBR is an effective treatment for TNBS-induced colitis. Treatment with BBR can reduce the pathological alterations and intestinal microbiota disturbance brought on by TNBS. This work is novel in that it shows that BBR can control some gut microbiota, and that *Akk* bacteria may play a significant role in the intestinal microbiota of BBR in mitigating colitis caused by TNBS (Zheng et al., 2023). It offers fresh theoretical justification for the future use of probiotics, symbolised by *Akk* bacteria, in the treatment of colitis.

According to Xu et al. (2023), *Akk* bacteria have the capacity to repair intestinal mucosal damage in mice, suppress inflammatory responses, relieve intestinal microbiota disorders, and modify the role of healthy intestinal microbiota. *Firmicutes* and *Bacteroidetes* are the principal microbes that make up the healthy gut microbiota. *Actinomycetes* and *Verrucomicrobia* came next. Furthermore, the vulnerability of illnesses is correlated with the ratio of *Firmicutes* to *Bacteroidetes*. Research has demonstrated that probiotics can control the diversity and quantity of intestinal microbiota, as well as the function of the intestinal barrier, antioxidant capacity, inflammatory cell factor secretion, immune function, production of antibacterial substances, inhibition of intestinal pathogen invasion and colonisation, and other aspects of colitis treatment (IBD).

Probiotics’ function in regulating immunity and metabolism has steadily come to light, as they play an increasingly significant role in preserving intestinal microecology and regulating intestinal flora. For instance, lactobacillus is involved in the treatment of severe metabolic illnesses, obesity, and diabetes, while *Akk* bacterium is involved in cancer, diabetes, and anti-aging. As a result, probiotics with the potential to treat a wide range of illnesses are worth researching, particularly those embodied by the *Akk* bacteria, which have few adverse effects in addition to their beneficial effects. In the future, the efficacy of probiotics in treating gastrointestinal tract disorders like colitis may be confirmed by the *Akk* bacterium. Probiotics are predicted to be mass-produced and extensively employed in the treatment of diseases in the future, while the molecular mechanism of their participation in specific diseases is still being investigated.

The use of a single mouse animal model, the lack of repeated studies, and the absence of clear causation evidence connecting particular microbiome changes to improvements in colitis are some of the drawbacks of this work. This work offers compelling *in vitro* data supporting BBR’s ability to modify the gut microbiota and alleviate colitis. Before using the medication in a therapeutic setting, more investigation should be done.

## 5 Conclusion

This study provides strong *in vitro* evidence that BBR can improve colitis by reshaping the gut microbiota. Further examination should be performed before clinical use of the drug.

## Data Availability

The original contributions presented in the study are publicly available. This data can be found here: https://www.ncbi.nlm.nih.gov/, accession number PRJNA1152768.
