# Supplementary material for: Berberine attenuates TNBS-induced colitis in mice by improving the intestinal microbiota
Source: Front Microbiol. 2024 Aug 29;15:1463005. doi: 10.3389/fmicb.2024.1463005 (PMC11392431; doi:10.3389/fmicb.2024.1463005)
Supplement: Supplementary file 1 [file Data_Sheet_1.PDF]

CD3（%）	Ctrl	TNBS	BBR
	38.8	76.9	43.1
	44.4	66.2	52.4
	38.3	71.4	59.2
	45.5	77.7	63.5
	38.9	82.2	61.3
	39.3	73.6	55.7
	43.7	74.7	57.3
Mean ±SD	41.27±3.11	74.67±5.07	56.07±6.78

CD4（%）	Ctrl	TNBS	BBR
	17.5	23.6	19
	19.5	27.1	19.4
	20.7	22.5	22.1
	19.4	26.8	22.3
	20.3	24.6	21.3
	18.8	25.7	19.2
	17.8	25.2	21.6
Mean ±SD	19.14±1.20	25.07±1.66	20.7±1.44

CD4/CD8	Ctrl	TNBS	BBR
	2.61	1.14	1.78
	2.03	1.20	1.66
	2.08	1.07	2.05
	2.14	1.31	1.74
	2.22	1.39	1.70
	2.85	0.94	1.85
	2.25	1.25	1.82
Mean ±SD	2.31±0.31	1.18±0.15	1.80±0. 13

CD8（%）	Ctrl	TNBS	BBR
	7.65	20.7	8.76
	8.43	19.6	11.7
	7.1	21.1	10.8
	9.07	19.3	12.8
	6.72	17.7	12.5
	6.59	20.4	14.9
	7.91	19.7	8.48
Mean ±SD	7.36±0.91	19.79±1.12	11.42±2.29

Th17（%）	Ctrl	TNBS	BBR
	2.5	6.4	1.8
	2.9	7.3	3.4
	3.7	5.2	4.2
	3.1	8.2	1.7

	2.8	6.8	2.8
	3.5	5.9	2.1
	3.4	6.3	2.5
Mean ±SD	3.12±0.43	6.58±0.97	2.64±0.91

Treg（%）	Ctrl	TNBS	BBR
	4.8	3.1	6.6
	4.3	4.4	5.6
	4.2	3.8	5.9
	4.9	5	6.2
	3.8	4.4	6.5
	4.1	4.7	7
	4.5	4.1	6.4
Mean ±SD	4.37±0.39	4.21±0.63	6.31±0.46

IL-17A	Ctrl	TNBS	BBR
	60.17	124.60	70.24
	74.26	108.49	96.41
	80.30	98.42	92.38
	48.09	80.30	64.20
	64.24	136.68	72.25
	58.10	102.45	80.30
Mean±SD	[illegible]4.19±11.6	108.49±19.9[illegible]	79.93±12.84

IL-4	Ctrl	TNBS	BBR
	73.80	125.61	99.70
	67.32	226.00	86.75
	64.08	158.00	77.03
	54.36	102.94	86.23
	70.56	109.42	70.56
	54.36	99.70	64.08
	73.80	80.27	89.99
Mean±SD	64.08±8.19	[illegible]36.95±48.5[illegible]	[illegible]0.72±12.81

IL-6	Ctrl	TNBS	BBR
	8.69	272.13	60.85
	5.14	192.62	85.04
	6.91	309.05	74.73
	13.12	181.01	88.52
	11.35	166.42	37.96
	6.03	189.66	62.79
	21.11	174.88	92.71
Mean±SD	8.54±3.14	[illegible]18.48±57.7[illegible]	[illegible]8.31±18.64

TNF-α	Ctrl	TNBS	BBR
	75.36	149.89	72.88
	67.91	125.05	115.11
	92.75	102.69	97.72
	75.36	120.08	95.24
	90.27	117.60	87.78
	75.36	110.14	75.36
	97.72	117.60	97.72
Mean±SD	79.50±9.77	[illegible]20.91±16.2[illegible]	[illegible]0.68±15.67

IFN-γ	Ctrl	TNBS	BBR
	12.04	35.45	12.91
	12.91	40.65	21.58
	15.51	34.59	18.98
	12.04	43.26	19.85
	12.91	39.79	13.78
	5.97	44.99	21.58

	12.91	45.86	24.18
Mean±SD	±3.17	39.79±4.14	18.11±3.84

TGF-β	Ctrl	TNBS	BBR
	3603.88	2176.40	3974.87
	2892.81	2336.33	3974.87
	2985.56	1625.26	3263.81
	2490.91	1810.75	4314.95
	2707.32	1965.33	3140.14
	3325.64	1934.42	4015.33
	3102.65	1996.25	3325.64
Mean±SD	01.02±406.	74.75±253.	80.66±467.63
