# Supplementary material for: Berberine attenuates TNBS-induced colitis in mice by improving the intestinal microbiota
Source: Front Microbiol. 2024 Aug 29;15:1463005. doi: 10.3389/fmicb.2024.1463005 (PMC11392431; doi:10.3389/fmicb.2024.1463005)
Supplement: Supplementary file 2 [file Table_1.DOCX]

# Supplementary Material：

Table 1：Spleen weight and colon length in each group

|  | Spleen weight（mg） | Colonic length（cm） |
| --- | --- | --- |
| Ctrl | 87.71±9.81 | 9.46±0.75 |
| TNBS | 60.29±6.70 | 6.79±0.53 |
| BBR | 120.71±12.46 | 8.36±0.48 |


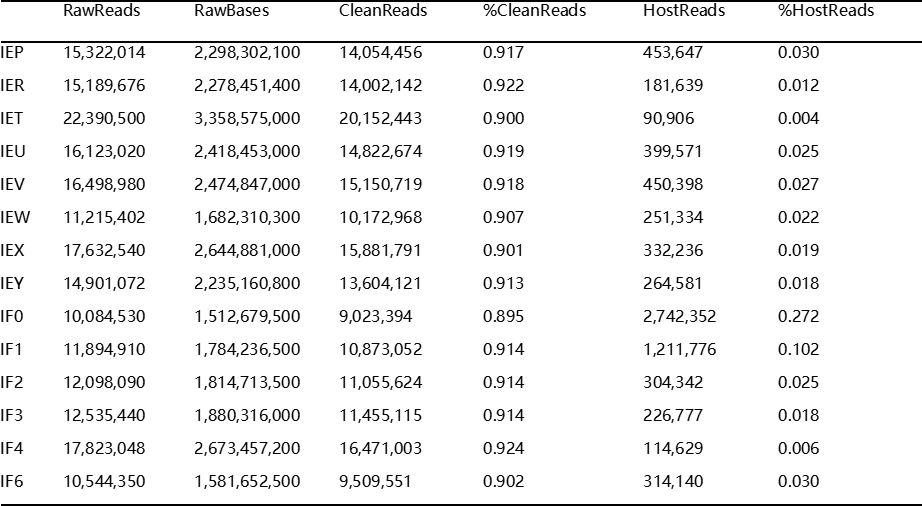

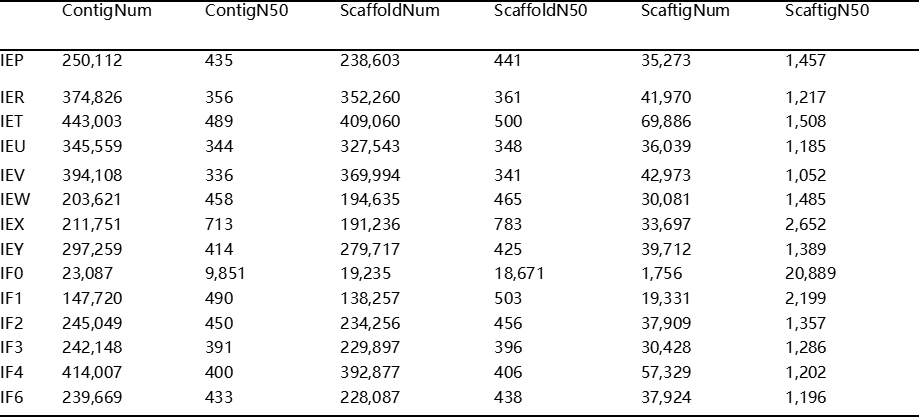


Table 2：Part of the sample sequencing basic statistics

Table 3: Part of the sample assembly results statistics
